# Size Influence of Laminated Bamboo on Tensile Mechanical Properties with the Consideration of Density

**DOI:** 10.3390/ma18020238

**Published:** 2025-01-08

**Authors:** Jiannan Li, Amardeep Singh, Rahul Sharma, Xinchen Yu, Junwen Zhou, Pei Ge, Shulan Yang

**Affiliations:** 1 School of Civil Engineering and Architecture, Changzhou Institute of Technology, Changzhou 213032, China; lijn@czu.cn (J.L.); zhoujw@czu.cn (J.Z.); gep@czu.cn (P.G.); yangsl@czu.cn (S.Y.); 2 School of Engineering, Design and Built Environment, Western Sydney University, Sydney, NSW 2751, Australia; 3 School of Civil Engineering, Shri Mata Vaishno Devi University, Katra 182320, India; 4 Hong Kong Huayi Design Consultants (Shenzhen) Ltd., Shenzhen 518000, China; yuxinchen@huayidesign.com

**Keywords:** laminated bamboo, tensile property, size effect, density influence, strength–modulus relationship

## Abstract

Laminated bamboo (LB), as a novel eco-friendly composite material with a high strength-to-weight ratio, has garnered increasing interest. However, there is a gap in comprehending the impact of size on the tensile properties of LB, particularly tensile strength and modulus. In contrast with conventional materials such as concrete and wood, which have specified specimen sizes and size effect factors to address performance variances linked to size, LB lacks such standards and references. To address this, seven groups of LB specimens of varying sizes were developed and tested to examine the impact of length, thickness, and sectional area on failure mechanisms and fundamental features, including density considerations. The findings demonstrate that size does not significantly influence the failure mode. Length exerts a smaller influence than thickness. When specimens are scaled up by a factor of two, tensile strength and modulus diminish to 82.86% and 88.38% of their initial values, respectively. Density significantly influences the relationships of tensile strength, modulus, and size effects. Consequently, size effect models incorporating both specific properties and the density–property relationship were developed.

## 1. Introduction

Bamboo is a natural composite material recognized for its superior strength-to-weight ratio and directional fiber reinforcement [1,2,3]. Known for its quick growth, minimal carbon emissions, and sustainability, bamboo has a long-standing history of utilization in building globally in its raw form. Nonetheless, obstacles associated with irregular sectional dimensions, intricate connections tailored to circular profiles, and the inconsistent arrangement of vascular bundles have impeded the advancement of contemporary large-scale bamboo constructions. To address these restrictions, various types of bamboo-based composites have been developed, one of which being laminated bamboo (LB). LB minimizes natural faults, standardizes dimensions, and enhances utilization efficiency through processes including cutting, splitting, grinding, antiseptic treatment, and hot-pressing. These methods improve performance and rectify the defects of raw bamboo, enabling its ultimate strength and modulus of elasticity parallel to the grain to reach up to 120 MPa and 12 GPa, respectively [4,5,6,7,8]. Consequently, LB offers the capability to serve as an alternative to wood and specific fiber-reinforced composite materials. 

Over the past decade, LB has been widely utilized in construction, especially for beams, columns, slabs, and wall components, and theoretical prediction methods grounded in nonlinear constitutive relationships have been established to simplify the design process [9,10,11,12,13]. Due to its exceptional mechanical properties, particularly its elevated tensile strength, composite components integrating LB with materials such as concrete [14,15,16,17,18], fiber-reinforced polymer (FRP) [19,20,21], and steel [22,23,24] have been suggested to enhance the load-bearing capacity while minimizing self-weight, capitalizing on bamboo’s superior strength-to-weight ratio. These investigations establish the basis for formulating design methodologies and extend to the computation of associated LB components. However, the intricate microstructure, heterogeneity, and numerous influencing elements of LB provide several unresolved issues, one of which pertains to size. Larger members typically experience failure at lower stress levels than smaller members, a phenomenon referred to as the size effect, which is true of nearly all materials. As a result, components of the same material that differ in size display inconsistent strength, which is likewise correlated with the size of the fibers and particles [25,26,27]. This issue, which has been noted in substances like concrete [28] and wood [29,30,31], is frequently managed by limiting specimen dimensions in testing protocols and incorporating size-correction variables in design regulations, although these parameters require thorough investigation for accurate determination.

As a kind of cellular and multi-layered material, LB demonstrates considerable size effect problems as a result of inherent and manufacturing faults [32]. Nonetheless, there exists a paucity of understanding in this domain. Numerous theories, including the weakest-link theory derived from Weibull’s statistical framework, have been employed to elucidate the size impact in wood-like materials [25,30,33,34] and concrete [35,36]. The weakest-link theory posits that as a structure’s size expands, the quantity of faults and flaws escalates, hence augmenting the probability of failure. However, this theory presupposes that structures are interconnected in series and exhibit linear behavior, warranting discussion on its applicability to LB. Furthermore, the weakest-link theory primarily elucidates the brittle failure mechanism, whereas LB is characterized as an elastic–plastic material exhibiting ductility and quasi-brittle behavior attributable to the presence of resin, intrinsic defects, and fiber bridges [37,38,39]. This leads to an appearance of brittleness during fiber fracture and yielding processes, although, in actuality, a ductile mechanism is at play. Theories of fracture energy and toughness may offer more suitable models for characterizing LB’s behavior. Bažant’s fracture energy size effect model, emphasizing the fracture process zone and energy release, indicates that smaller specimens exhibit more ductility, whereas larger specimens demonstrate increased brittleness. This model has exhibited significant accuracy and adaptability for quasi-brittle materials, including concrete [40,41,42]. Furthermore, Carpinteri’s multi-fractal theory, which characterizes materials as disordered, has been employed to elucidate size effects stemming from the stochastic and discontinuous characteristics of fractures [43,44,45,46]. Diverse methodologies, such as regression analysis, likelihood theory, reliability theory, cohesive models, multi-scale modeling, and the extended/discrete element method, have been utilized to investigate size impacts [34,47,48,49,50,51,52]. Cohesive models use pairing parts to depict how two surfaces of a possible fracture interact. The crack starts to spread when the cohesive force between the connected parts is eliminated. Cracks can freely pick their propagation direction when simulating using the extended/discrete element approach, which does not prescribe the crack propagation path. However, the LB constitutive model has not been developed, so it is not mature, and the aforementioned approaches depend on a thorough understanding of the material, such as the fracture process zone and fracture toughness, which are currently poorly understood. Nonetheless, because of LB’s considerable orthotropic nature, additional examination is required of the variation in failure mode and mechanical properties with size.

Typically, the fundamental strength correlates with specimen dimensions, leading to particular criteria and size effect factors in testing standards. In contrast to concrete, wood products possess several design requirements, such as the NDS [53], CSA O86 [54], AS 1720.1 [55], and GB 50005 [56], each offering distinct methodologies, dimensions, or configurations. Unifying test findings from varying sizes is challenging, and it is significantly more arduous to establish a benchmark due to the absence of a reference. Furthermore, when materials are used in other regions, they must be reassessed in accordance with local criteria to substitute factory performance, complicating their application. Consequently, comprehending the size impact is essential for achieving mechanical performance.

This work seeks to address this knowledge gap by investigating the impact of size on LB’s tensile performance. Seven specimen groups of varying sizes were examined and analyzed to examine the impact of length, thickness, and cross-sectional area on failure mechanisms and fundamental mechanical parameters. Weibull’s statistical theory, Bažant’s fracture energy model, and Carpinteri’s multi-fractal scaling law were used to explicate the observed phenomena and develop efficient estimate approaches. The influence of density on size effects was also examined. Ultimately, two novel methods were developed—one grounded in specific properties and the other in the correlation between density and mechanical qualities. These methodologies were used to get more precise forecasts and establish the size effect law.

## 2. Materials and Methods

### 2.1. Materials

**Figure 1:**
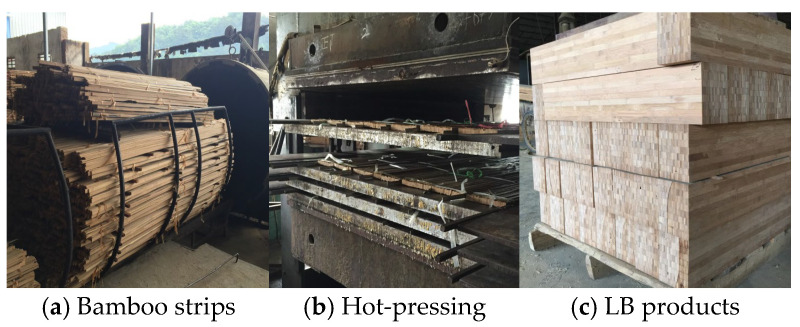
Manufacturing process of LB.

Laminated bamboo (LB) is manufactured from raw bamboo, particularly from bamboo aged three to five years, obtained from authorized production regions. The collected bamboo culms are further chopped, graded, and screened, yielding sections of varied lengths, often ranging from 1 to 2 m. The sections are then divided and cut into homogeneous bamboo strips in the radial direction and subsequently ground to eliminate the internodes and outer layers, as shown in Figure 1a. Bamboo strips are submerged in an antiseptic solution for mold protection and then kiln-dried at 80 °C until an average moisture content of 11% is attained, ensuring resistance to mold and insects while enhancing longevity. A handheld moisture content tester is used for real-time evaluation of the moisture content. The processed bamboo strips are uniformly coated with phenolic resin, arranged in layers according to design parameters, and subjected to heat pressing. The phenolic resin has an elastic modulus of 2575 MPa and a Poisson’s ratio of 0.42, with a density of 300 g/m^2^. The hot-pressing procedure occurs at a temperature of 100–110 °C, with a pressure of 15–20 MPa, for a period of 10–20 min, facilitating the melting of the resin to occupy the interstices between the strips, as shown in Figure 1b. Subsequent to these processing procedures, the unembellished laminated bamboo material is acquired (Figure 1c).

**Figure 2:**
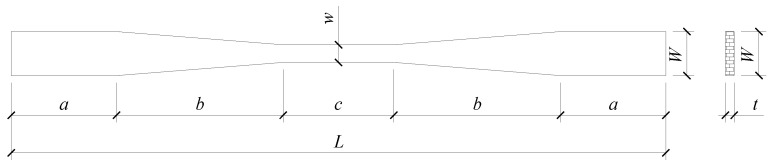
Sample dimension diagram.

The samples were then chopped and polished by Fujian Xinda Bamboo Company. Figure 2 illustrates the dimensions. Each group included 20 samples, with their dimensions specified in Table 1. Groups L1 was the standard group, the dimensions of which were determined by ASTM D143 [57]. Groups L1, L6, and L7 were established to explore the impact of proportional scaling, groups L2–L3 and L5–L6 to assess the effect of transition length *b*, specifically the variance in tapered transition lengths, and groups L2–L7 to evaluate the effects of thickness *t*.

The density and moisture content (MC) of laminated bamboo were evaluated in accordance with GB/T 1927.4 [58] and GB/T 1927.5 [59]. The results are shown in Table 2, where the coefficient of variation (CV) is determined by dividing the standard deviation (SD) by the mean value.

### 2.2. Loading and Measurement Methods

The loading schedule was established according to ASTM D143 [57] and GB/T 1927.9 [60]. The specimens underwent a loading rate of 2 mm/min until failure, preceded by six loading and unloading cycles between 10% and 30% of the expected ultimate load to assess the elastic characteristics. Specimens in groups L4 and L7 were evaluated using Wance 200-ton hydraulic testing equipment due to size and loading capacity constraints, whilst all other groups were assessed using a Sans 5-ton hydraulic testing machine.

**Figure 3:**
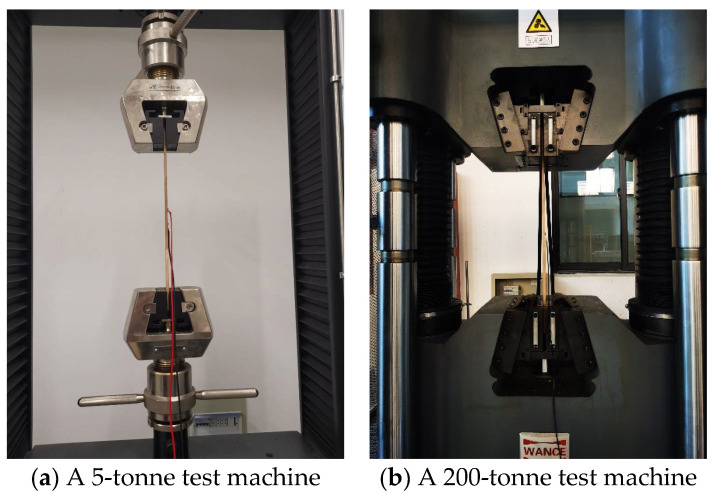
Test machines and set-up.

Figure 3 demonstrates that two aligned fixtures were used on each side of the specimens to replicate uniaxial tensile conditions. Strain and displacement data were acquired using the TDS-530 data collection system, with two strain gauges located at the midpoint along the loading direction and perpendicular to the grain to measure tensile strain and Poisson’s ratio, respectively. The strain gauge measured 5 mm × 3 mm, had a resistance of 120.1 Ω, and exhibited a gauge factor of 2.10.

According to related standards mentioned above, the tensile strength $f_{t,1}$ can be calculated as follows:

(1) $$f_{t,1}=\frac{F_{u}}{w\cdot t}$$

where $F_{u}$ is the ultimate capacity, N, and w and t are sectional width and thickness, respectively, mm. The tensile modulus $E_{t,1}$ can be calculated from the last four loading and unloading cycles:

(2) $$E_{t,1}=\frac{\Delta \sigma }{\Delta \varepsilon }$$

where Δσ is the change in tensile stress, MPa, and Δε is of the change in tensile strain, ε. The Poisson’s ratio $\gamma_{21}$ can also be calculated from the last four loading and unloading cycles:

(3) $$\gamma_{21}=-\frac{\Delta \varepsilon_{2}}{\Delta \varepsilon_{1}}$$

where $\Delta \varepsilon_{1}$ and $\Delta \varepsilon_{2}$ are the change in tensile strain parallel to grain and perpendicular to grain, respectively, ε.

## 3. Results and Discussion

### 3.1. Test Results

**Figure 4:**
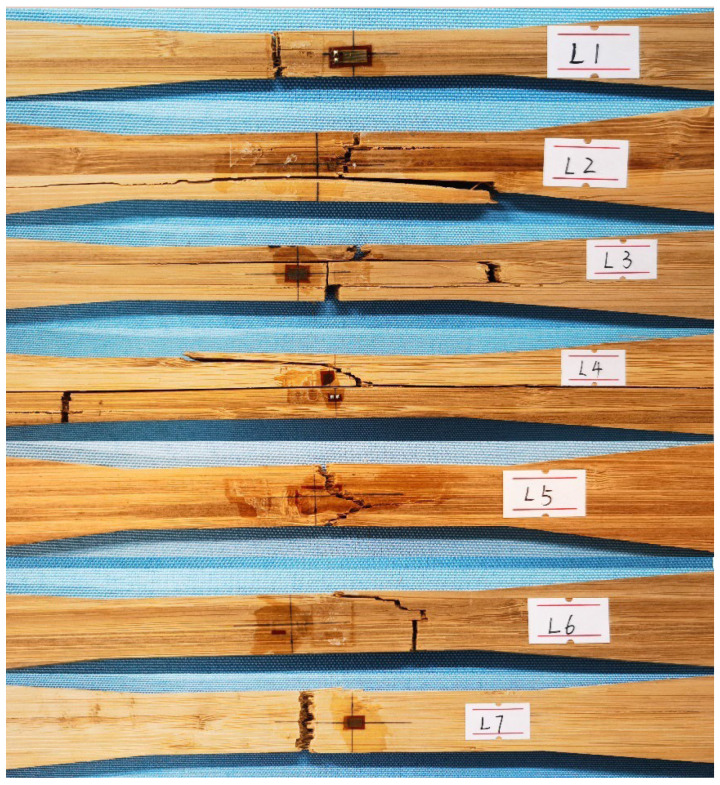
Typical failure modes.

In the first loading stage, the specimen mostly exhibited elastic behavior, with deformation progressively rising. Occasional cracking noises were produced as the pressure intensified, and minor surface fractures were noted on many specimens, leading to a small reduction in load capacity. Upon surpassing the imposed load threshold, substantial fractures manifested abruptly and swiftly in the central effective zone, indicative of failure. Figure 4 illustrates the standard failure mechanisms for each category.

Analysis of the failure sections indicated that the predominant damage resulted from the rupture of bamboo fibers, but a minor fraction was attributed to shear failure between bamboo strips, as shown in groups L2, L3, and L4. The heterogeneous composition of LB material leads to variations in the elastic modulus of individual bamboo strips, causing inconsistent deformation and shear stress among layers under uniform force. Consequently, interlayer separation may transpire under substantial stresses. Analysis of the diverse failure mechanisms across each group reveals that as size escalates, the damaged area becomes more irregular, attributable to the growing number of bamboo strips engaged in load bearing. The alterations in size hardly affect the failure mode, since all failures are tensile in the center effective zone.

The final findings are provided in Table 3, using Equations (1)–(3) and the Grubbs test. The low coefficient of variation validated the precision and representativeness of the data. The tensile strength varied from 73.22 MPa to 113.78 MPa, with the peak limit being 1.55 times larger than the lower limit. In comparison to the L1 group, designated as the control group with conventional dimensions, tensile strength values varied between 64.35% and 96.40% of the strength of the L1 group. The fluctuations in tensile modulus and strain were minimal, with tensile modulus values spanning from 87.43% to 105.73% and strain values from 78.74% to 94.68% in comparison with the L1 group. The Poisson’s ratio stayed essentially constant.

Notwithstanding these minor variances, it is clear that the findings, including tensile strength, modulus, and strain, especially for groups with substantial size differences, exhibited considerable discrepancies, thus offering compelling evidence for the existence of a size effect phenomenon.

### 3.2. Size Effect Analysis

#### 3.2.1. Single Size Effect

**Figure 5:**
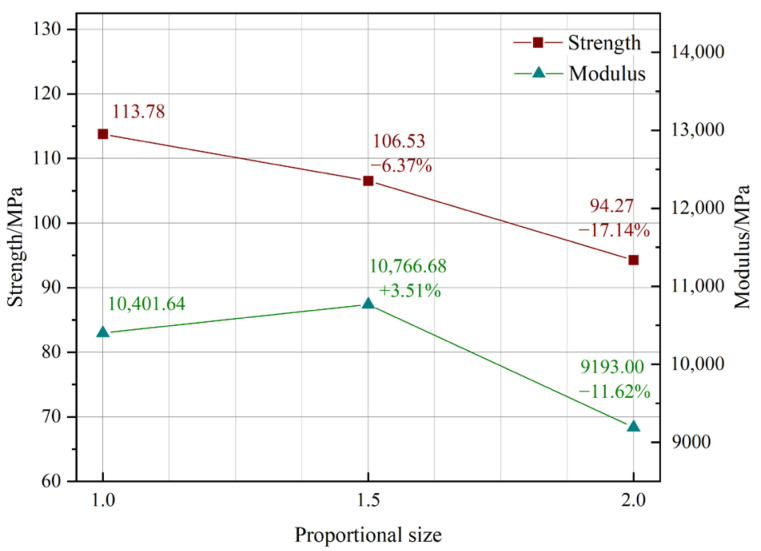
Proportional size effect.

This research largely concentrated on the analysis of strength and modulus, owing to their significance in the application of tensile components. The findings from groups L1, L6, and L7 demonstrated that both strength and modulus dramatically decreased with a proportionate increase in specimen size, as seen in Figure 5. The correlation between specimen size and strength was almost linear; however, minor deviations were seen in the modulus curves. When the proportionate ratio was 1.5, there was little variation in strength and modulus. As the size increased, the decline became more significant, although the extent was less than 20%. The tensile size impact is likely to be moderate.

Upon doubling the specimen size compared to the normal size (corresponding to quadrupling the standard sectional area), the strength and modulus decreased to 82.86% and 88.38% of the standard specimen, respectively. The main reason for this drop is the heightened presence of strips and glue lines, which resulted in faults such as bubbles in the adhesive layer, insufficient compactness, and pre-existing fissures in the original bamboo, hence elevating the risk of damage.

**Figure 6:**
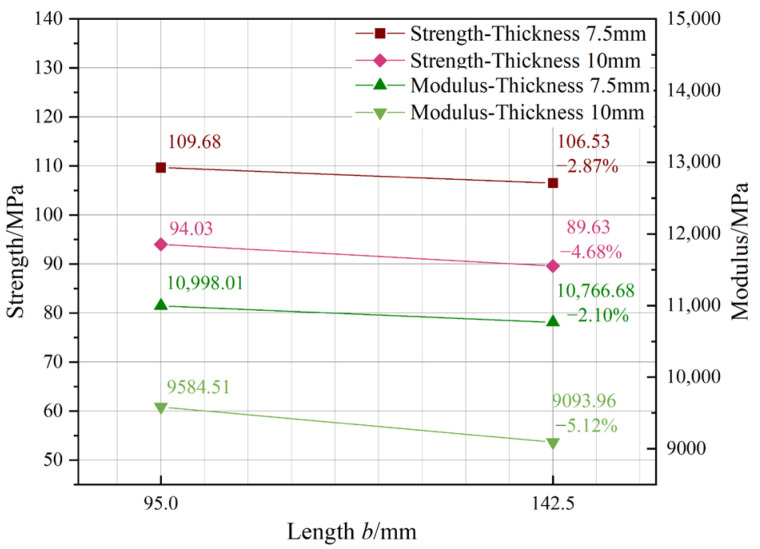
Length *b* influence.

Figure 6 depicts the impact of length *b*. The graph demonstrates that both modulus and strength decreased with increasing length. In comparison to the conventional L1 group, the strength and modulus were reduced by a maximum of 4.68% and 5.12%, respectively. The reduction ratio had a less apparent size impact. The decrease does not greatly change with thickness, indicating that length *b* is not a primary factor in the size impact.

**Figure 7:**
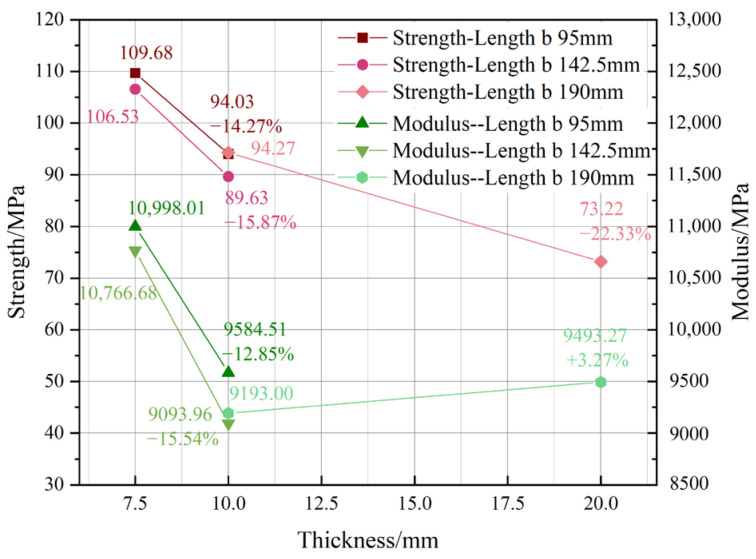
Thickness influence.

Figure 7 depicts the changes in modulus and strength due to modifications in thickness dimensions for specimen transition lengths of 94 mm, 142.5 mm, and 190 mm, respectively. As the thickness rose, most attributes diminished, although at a very minor pace. A marginal rise in modulus was seen with a length of 190 mm; nevertheless, this does not provide proof of a positive association between modulus and thickness. The trends of strength and modulus with lengths of 95 mm and 142.5 mm were very similar, indicating that the effect of length b on the performance–thickness relationship may be disregarded. The impact on strength was more pronounced than that on modulus, particularly with increased thickness. This suggests that the influence of internal defects in bamboo strips surpasses that of adhesive layer issues.

**Figure 8:**
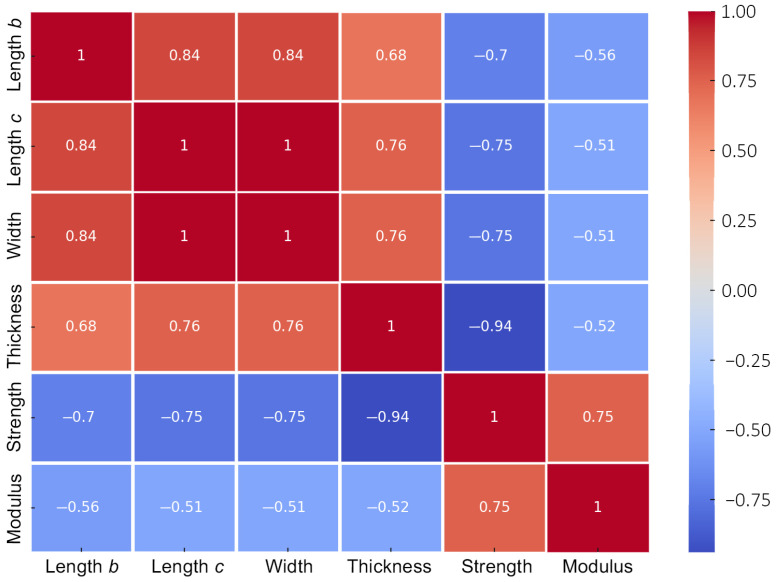
Correlation heatmap.

Figure 8 illustrates the relationship between size dimensions and mechanical characteristics derived from Equation (4). The red regions signify robust positive correlations, indicating that a rise in one measure corresponds with an increase in the other. The blue regions signify negative associations. Consequently, thickness has a significant negative connection with strength, indicating that increased thickness generally reduces strength. The width, as well as lengths b and c, demonstrate a small but significant negative connection, which may be regarded as a manifestation of the size effect. A robust positive connection exists between modulus and strength, which may serve as a reference for determining strength from modulus in non-destructive testing.

(4) $$r=\frac{\sum \left(x_{i}-\bar{x}\right)\left(y_{i}-\bar{y}\right)}{\sqrt{\sum {\left(x_{i}-\bar{x}\right)}^{2}\cdot \sum {\left(y_{i}-\bar{y}\right)}^{2}}}$$

where r is the correlation factor, and $\bar{x}$ and $\bar{y}$ are the mean values of the x and y data sets, respectively.

#### 3.2.2. Sectional Area Size Effect Analysis

Table 4 illustrates the variations in modulus, strength, and strain corresponding to different sectional areas. The findings demonstrate that strain diminishes as sectional area increases, ranging from 1.10% to 0.87%. In the majority of cases, the strength diminishes as the cross-sectional area increases, varying from 113.78 MPa to 73.22 MPa. The range of variation for the modulus and Poisson’s ratio is minimal and lacks a discernible pattern of change.

**Figure 9:**
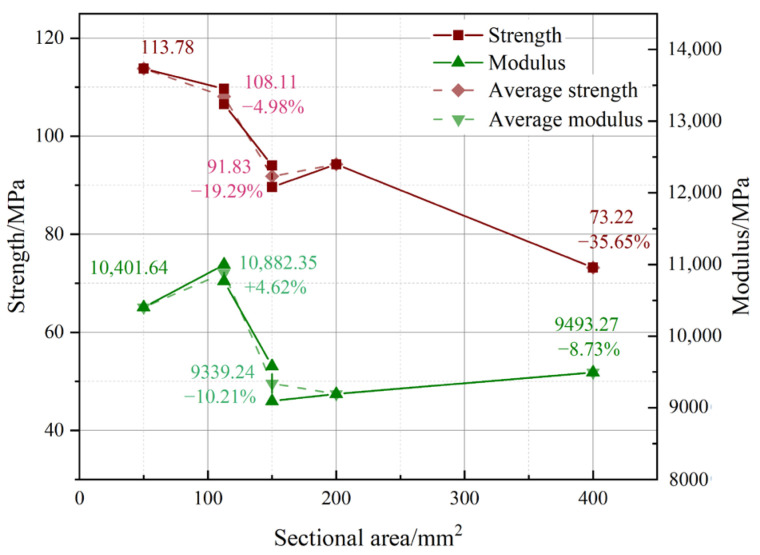
Influence of sectional area on strength and modulus.

Figure 9 illustrates that both modulus and strength decrease as the sectional area increases, with a more significant impact seen in smaller sizes, while bigger sizes seem to stabilize. When the sectional area reached 400 mm², the strength and modulus were just 64.35% and 91.27% of the conventional size, respectively. The average strength and modulus of identical areas exhibit analogous tendencies, indicating that sectional area is a crucial aspect to consider as a size control index when evaluating the size impact.

### 3.3. Size Effect Models

Three typical size effect models, based on the weakest-link [61], fracture [62], and fractal theories [63], were considered in this study. The formulas were expressed as follows and named as method 1:

(5) $$M_{N,Wbl}=A_{1}D^{-B_{1}}$$

(6) $$M_{N,Fru}=B_{2}f_{t}\cdot {\left(1+D/D_{0}\right)}^{-1/2}$$

(7) $$M_{N,Frt}=\sqrt{A_{3}+B_{3}/D}$$

where *D* is the size effect index representing single size, area, or volume; *A*_1_, *B*_1_, *B*_2_, *D*_0_, *A*_3_, and *B*_3_ are parameters which could be determined by curve fitting; *f*_t_ is the tensile strength and is 113.78 MPa in this study; and *M* is the mechanical properties, which could be tensile modulus or tensile strength.

#### 3.3.1. Single Size Influence

Nonlinear curve fitting was used to ascertain the parameters, as seen in Table 5. The examination of the sum of squares of deviations (SSE) demonstrates that the fracture model is the most appropriate for precisely depicting the data. The estimated results derived from the suggested size effect models are shown in Table 6. In comparison with the experimental results, the differences and mistakes were negligible, illustrating the efficacy of this strategy, but it is only relevant when dimensions increase correspondingly. Furthermore, using just three data points for fitting may limit the model’s applicability to other data sets.

#### 3.3.2. Thickness and Area Size Effect Analysis

To improve the precision of the descriptive models, both thickness and cross-sectional area were used to signify the impact of size. The data from groups L1 to L7 were used, and the model parameters are shown in Table 7. The findings indicate that, irrespective of the usage of thickness or area, the optimum model for strength was the fractural model. This signifies that damage initiates from early microcracks, and when energy is dissipated, the damage propagates along the crack route, leading to irregular fracture surfaces, in accordance with the failure mechanism shown in Figure 3.

The estimated values are shown in Table 8 and Table 9, using the optimal models. All statistics indicate quite small errors, below 10%. The projected values derived from thickness exhibited more accuracy than those based on cross-sectional area. Despite the mistakes being more than those of proportionate groups, the model’s applicability has expanded. Both techniques exhibited minimal average prediction errors and commendable predictive accuracy.

### 3.4. Influence of Density

#### 3.4.1. Relationship of Modulus, Strength, and Density

**Figure 10:**
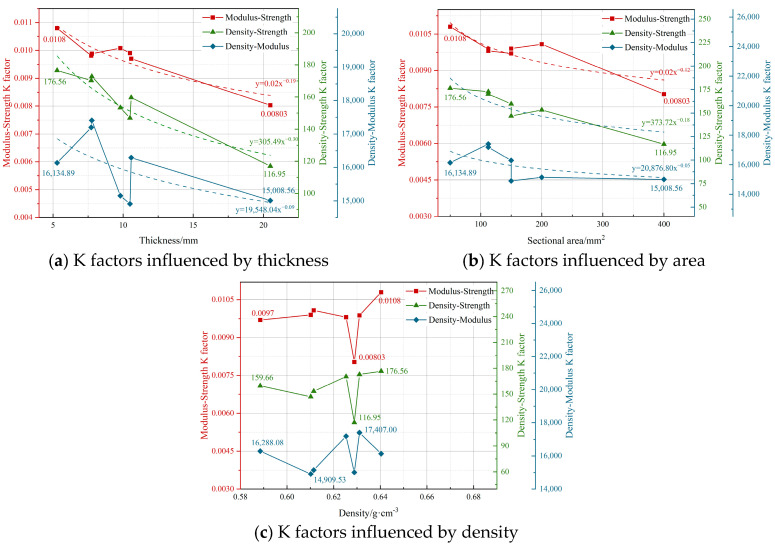
Relationships of modulus, strength, and density.

To enable the estimation of mechanical properties, straightforward correlations were constructed among modulus, strength, and density. The relationships among modulus–strength, density–strength, and density–modulus were analyzed, with trends affected by thickness, sectional area, and density, as seen in Figure 10. The variables decreased as thickness and sectional area rose, whereas no significant variations were seen with changes in density. Consequently, the size effect has a more significant impact on the correlations among modulus, strength, and density than density by itself. When carrying out non-destructive testing or empirical calculations, the influence of size effects must be meticulously evaluated, and suitable modifications should be implemented.

#### 3.4.2. Single Size Effects

Table 10 presents the specific modulus and specific strength results from the tests. A pronounced downward trend indicates that these specific properties were markedly affected by size. The range of fluctuation for specific strength exceeded that of specific modulus, fluctuating from 115.93 × 10^−3^ MPa·kg^−1^·m^3^ to 178.75× 10^−3^ MPa·kg^−1^·m^3^. The very low standard deviation and coefficient of variation indicate that this significant fluctuation was not a result of data dispersion.

**Figure 11:**
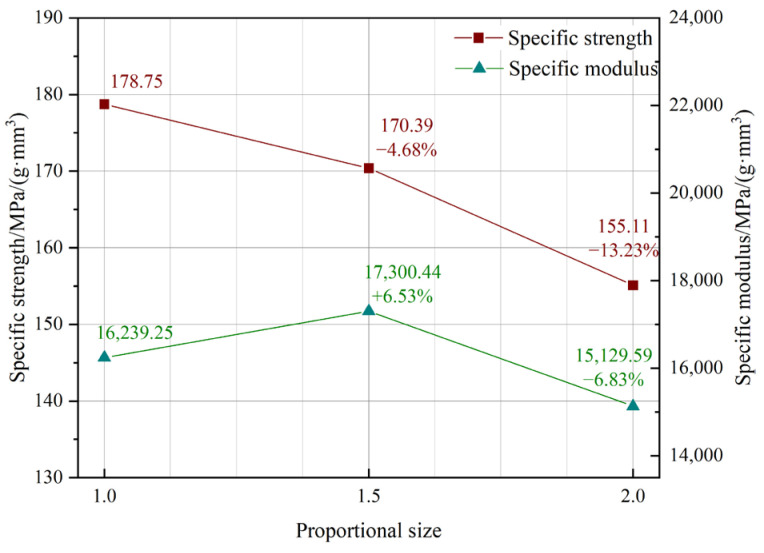
Proportional size effect on specific properties.

**Figure 12:**
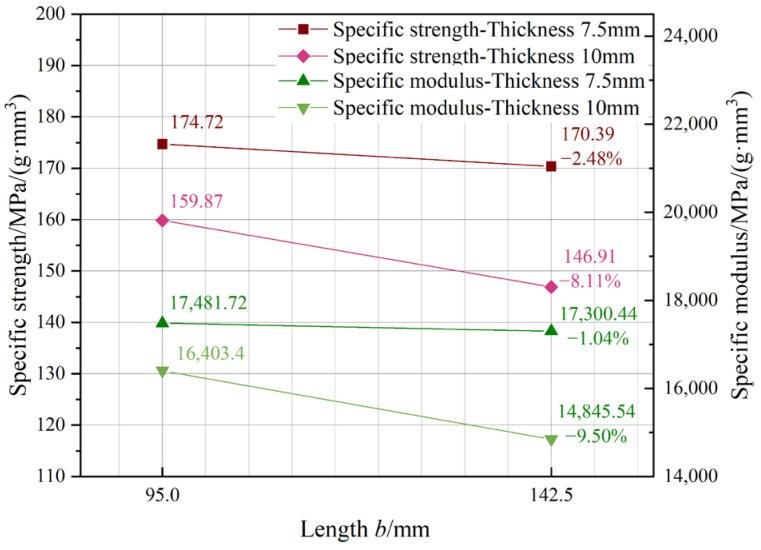
Length *b* influence on specific properties.

Figure 11 illustrates that, under proportionate magnification, both specific strength and specific modulus diminish to differing extents, with the reduction in specific strength occurring at a comparatively greater pace. When the side length is doubled and quadrupled relative to the initial length, the specific strength diminishes by 4.68% and 13.23%, respectively, corresponding to 95.32% and 86.77% of the specific strength of the standard specimen. When the side length doubles, the reduction does not display a distinct stabilizing pattern, suggesting that the particular strength may continue to decline, similar to the observed trend in strength. Conversely, each group had smaller variations than the general properties, indicating the impact of density on the size effect. Figure 12 demonstrates that, when accounting for the effect of length *b*, specific strength displays a similar trend to the general attributes, marked by a consistent decline. The initial reduction is slow, followed by a more precipitous fall.

**Figure 13:**
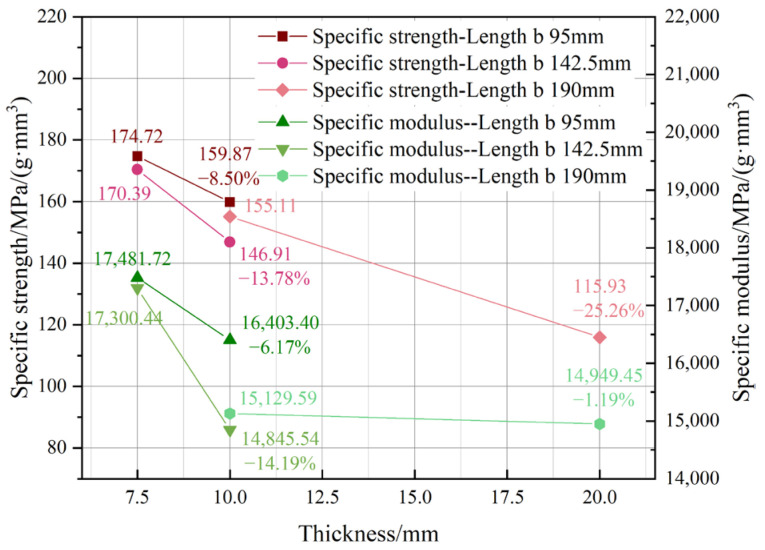
Thickness influence on specific properties.

Figure 13 illustrates the influence of thickness. The tendencies resemble those of general attributes, exhibiting a negative connection between performance and size. The specific strength and modulus decrease more quickly for transition lengths of 95 mm and 142.5 mm. In relation to general qualities, the reduction ratios are diminished, indicating that density has an impact on the size effect. The mechanical qualities of individual bamboo strips, from which LB is derived, are not fully homogeneous, hence influencing the overall characteristics of LB products. This research examined the impact of density on mechanical characteristics and used density to refine the projected outcomes, given the direct correlation between attributes and density. The specific strength and specific modulus were determined by dividing the strength and modulus by density to mitigate the impact of density.

#### 3.4.3. Size Effect Models

Based on the same theories, three size effect models are used as follows and named as method 2:

(8) $$M_{N,Wbl}=A_{1}D^{-B_{1}}$$

(9) $$M_{N,Fru}=B_{2}f_{t}\cdot {\left(1+D/D_{0}\right)}^{-1/2}$$

(10) $$M_{N,Frt}=\sqrt{A_{3}+B_{3}/D}$$

where *M* is the mechanical properties, which could be specific tensile modulus or specific tensile strength. The other parameters are the same as in Equations (5)–(7). Considering the relationship between tensile modulus, tensile strength, and density, the size effect models can be written as follows and named as method 3:

(11) $$M_{N,Wbl}=A_{1}D^{-B_{1}}\cdot \rho$$

(12) $$M_{N,Fru}=B_{2}f_{t}\cdot {\left(1+D/D_{0}\right)}^{-1/2}\cdot \rho$$

(13) $$M_{N,Frt}=\sqrt{A_{3}+B_{3}/D}\cdot \rho$$

where *ρ* is the average density of each group of valid data. The other parameters are the same as in Equations (5)–(10). The parameters were derived using the equations below and are shown in Table 11 and Table 12, respectively. The fractal model emerged as the most appropriate for the optimal fitting of specific strength using method 1 with area as the dimensional index, but the SSE for the weakest-link model was somewhat greater. In other circumstances, the most suitable model type was typically the weakest-link model. This indicates that the weakest-link hypothesis is better suited for elucidating certain qualities than the other two theories.

Table 13 and Table 14 provide the estimate findings together with their associated errors. In comparison to Table 8 and Table 9, it is apparent that the strength errors utilizing approach 3 are smaller than those derived from approaches 1 and 2; however, the modulus errors are greater. Moreover, using thickness as the dimensional parameter significantly enhanced predictive accuracy. The investigation indicated that methods 1 and 3 provide comparatively good forecast accuracy for modulus and strength, respectively.

It should be mentioned that while the optimal approach has the smallest average error, this does not imply that the prediction error for each size is the minimum. It is clear that method 2 is more accurate as the elastic modulus errors of the W5 group, which were determined using methods 1, 2, and 3 with thickness as the dimension index, are −8.01%, −6.15%, and −6.45%, respectively. Individual variations should thus be taken into account in practical applications, and individual and general faults should be investigated independently in order to choose the best approach.

## 4. Conclusions

This research established seven sets of LB tensile tests with varying specimen sizes to examine the size influence on tensile performance. Uniaxial tensile tests were performed to gather data on tensile strength, modulus, and strain, and to examine the impact of size variations on failure mechanisms and mechanical parameters. The impact of several size factors was analyzed, including single-sided dimensions, length, thickness, and cross-sectional area. Three prediction models for modulus and tensile strength were developed based on the weakest-link theory, fracture mechanics, and fractal theory, taking into account the impact of density.

1. Variations in size did not significantly influence the failure mechanism, as all specimens failed near the central midpoint, mostly exhibiting brittle failure attributed to fiber cracking. No significant link existed between failure mechanism and specimen dimensions; nevertheless, as size increased, the damaged section became progressively more uneven due to material inhomogeneity.
2. In most instances, proportionate expansion, characterized by an increase in length *b* and thickness, resulted in reductions in tensile strength and modulus. Nonetheless, the total alteration is rather small, and length *b* was not a key determinant influencing changes in mechanical properties, as corroborated by correlation analysis. Upon scaling by a factor of two, strength and modulus decreased to 82.86% and 88.38% of their original values, respectively. Variations in size exerted a more significant influence on strength than on modulus. Augmentations in cross-sectional area resulted in diminutions in modulus, strength, and strain, with smaller specimens demonstrating more pronounced reductions. No size effect phenomenon exists in Poisson’s ratio.
3. Size effect models for modulus and strength were developed based on three hypotheses about proportionate single size, thickness, and cross-sectional area. The comparison of calculated values with experimental values indicated that the model for proportional size changes exhibited greater accuracy, with average errors of 0.07% for strength and 0.27% for modulus. This concept is only relevant to proportional scaling. The models developed based on thickness and cross-sectional area exhibited considerable accuracy, with the thickness model achieving the maximum precision, resulting in average errors of 0.15% for strength and 0.32% for modulus.
4. The investigation into the impacts of density and size on the connections among modulus, strength, and density revealed that size affects all three relationships. As size increases, the correlation coefficients diminish, although the declining tendency becomes less pronounced. Consequently, size effects are pertinent in the correlations among modulus, strength, and density, necessitating attention in empirical assessments. The impact of density on these interactions was shown to be minimal and may be mostly disregarded.
5. The specific strength and modulus were analyzed concerning variations in particular size dimensions, demonstrating tendencies aligned with general features while displaying discrepancies attributable to density. Two types of size effect prediction models were presented based on the correlation between particular attributes and the modulus/strength–density relationship. The results indicated that the model using specified properties had great predictive accuracy, with thickness as the size index yielding reduced errors. Methods 1 and 3 were used to estimate modulus and strength in order to obtain high accuracy findings.

In future research, more parameters ought to be investigated because there are not enough experimental groups. The density and strength gradient should be increased as much as feasible in order to improve the formula’s applicability. The influence of moisture content and adhesive properties should also be considered to strengthen engineering applications.

## Figures and Tables

**Table 1:** Dimensions of samples.

Group Number	*w*/mm	*W*/mm	*t*/mm	*a*/mm	*b*/mm	*c*/mm	*L*/mm
L1	10	25	5	60	95	63	373
L2	15	37.5	10	60	95	94.5	404.5
L3	15	37.5	10	90	142.5	94.5	559.5
L4	20	50	20	120	190	126	746
L5	15	37.5	7.5	60	95	94.5	404.5
L6	15	37.5	7.5	90	142.5	94.5	559.5
L7	20	50	10	120	120	126	746

**Table 2:** Density and MC test results.

Statistical Parameters	Air-Dried Density *ρ*_ad_/g·cm^−3^	Oven-Dried Density *ρ*_od_/g·cm^−3^	MC
Mean value	0.62	0.60	7.63%
Standard deviation	0.03	0.03	0.19%
CV	4.14%	4.36%	2.55%

**Table 3:** Tensile tests results.

Group Number	Statistical Parameters	$\mathrm{Ultimate}\;\mathrm{Strength}\;f_{t,1}$/MPa	$\mathrm{Tensile}\;\mathrm{Modulus}\;E_{t,1}$/MPa	$\mathrm{Strain}\;\varepsilon_{t,1}$	$\mathrm{Poisson}’s\;\mathrm{Ratio}\;\gamma_{21}$
L1	Mean value	113.78	10,401.64	1.10%	0.26
	Standard deviation	11.57	1878.20	0.22%	0.02
	CV	10.17%	18.06%	20.03%	5.80%
L2	Mean value	94.03	9584.51	1.03%	0.25
	Standard deviation	16.62	1524.60	0.19%	0.06
	CV	17.68%	15.91%	18.06%	22.84%
L3	Mean value	89.63	9093.96	1.04%	0.24
	Standard deviation	20.88	945.51	0.26%	0.04
	CV	23.29%	10.40%	24.71%	18.46%
L4	Mean value	73.22	9493.27	0.87%	0.26
	Standard deviation	19.28	891.25	0.23%	0.04
	CV	26.33%	9.39%	26.13%	14.45%
L5	Mean value	109.68	10,998.01	1.02%	0.28
	Standard deviation	16.18	1227.82	0.17%	0.04
	CV	14.76%	11.16%	16.75%	15.24%
L6	Mean value	106.53	10,766.68	1.00%	0.25
	Standard deviation	10.54	554.82	0.14%	0.03
	CV	9.89%	5.15%	13.86%	13.48%
L7	Mean value	94.27	9193.00	1.02%	0.22
	Standard deviation	9.66	1283.22	0.10%	0.02
	CV	10.24%	13.96%	10.30%	9.46%

**Table 4:** The mechanical properties varied with sectional area.

Group Number	Sectional Area/mm^2^	$\mathrm{Ultimate}\;\mathrm{Strength}f_{t,1}$/MPa	$\mathrm{Tensile}\;\mathrm{Modulus}E_{t,1}$/MPa	$\mathrm{Strain}\varepsilon_{t,1}$	Poisson’s Ratio $\gamma_{21}$
L1	50	113.78	10,401.64	1.10%	0.26
L5	112.5	109.68	10,998.01	1.02%	0.28
L6	112.5	106.53	10,766.68	1.00%	0.25
L2	150	94.03	9584.51	1.03%	0.25
L3	150	89.63	9093.96	1.04%	0.24
L7	200	94.27	9193.00	1.02%	0.22
L4	400	73.22	9493.27	0.87%	0.26

**Table 5:** Parameters of size effect models based on proportional size.

Properties	Weakest-Link Model			Fractural Model			Fractal Theory Model		
	*A*_1_	*B*_1_	SSE	*B*_2_ *f*_t_	*D*_0_	SSE	*A*_3_	*B*_3_	SSE
Strength	207.60	0.26	13.80	152.20	13.16	9.67	5362.00	7.88 × 10^4^	18.66
Modulus	1.51 × 10^4^	0.15	7.78 × 10^5^	1.23 × 10^4^	31.24	6.91 × 10^5^	7.34 × 10^7^	4.04 × 10^8^	8.65 × 10^5^

**Table 6:** Comparison of estimated values and experimental values based on proportional size.

Group Number	Strength			Modulus		
	Estimated Value/MPa	Experimental Value/MPa	Error/%	Estimated Value/MPa	Experimental Value/MPa	Error/%
L1	114.73	113.78	0.84%	10,687.95	10,401.64	2.75%
L6	104.05	106.53	−2.33%	10,093.57	10,766.68	−6.25%
L7	95.88	94.27	1.71%	9588.47	9193.00	4.30%
Average			0.07%			0.27%

**Table 7:** Parameters of size effect models based on thickness and area.

Properties	*D* Index	Weakest-Link Model			Fractural Model			Fractal Theory Model		
		A_1_	B_1_	SSE	B_2_	D_0_	SSE	A_3_	B_3_	SSE
Strength	Thickness	201.40	0.33	90.72	168.10	4.64	73.64	2898.00	5.76 × 10^4^	121.80
	Area	261.20	0.20	209.70	130.40	188.10	139.70	5866.00	4.44 × 10^5^	370.90
Modulus	Thickness	1.28 × 10^4^	0.113	2.31 × 10^6^	9.93 × 10^3^	−2.40 × 10^7^	3.62 × 10^6^	7.45 × 10^7^	2.09 × 10^8^	2.24 × 10^6^
	Area	1.42 × 10^4^	0.072	2.40 × 10^6^	1.07 × 10^4^	1018.00	2.59 × 10^6^	8.61 × 10^7^	1.52 × 10^9^	2.61 × 10^6^

**Table 8:** Comparison of estimated values and experimental values based on thickness.

Group Number	Strength			Modulus		
	Estimated Value/MPa	Experimental Value/MPa	Error/%	Estimated Value/MPa	Experimental Value/MPa	Error/%
W1	116.62	113.78	2.50%	10,783.32	10,401.64	3.67%
W2	94.64	94.03	0.64%	9766.27	9584.51	1.90%
W3	94.64	89.63	5.59%	9766.27	9093.96	7.39%
W4	72.95	73.22	−0.37%	9215.75	9493.27	−2.92%
W5	103.92	109.68	−5.25%	10,116.65	10,998.01	−8.01%
W6	103.92	106.53	−2.45%	10,116.65	10,766.68	−6.04%
W7	94.64	94.27	0.38%	9766.27	9193.00	6.24%
Average			0.15%			0.32%

**Table 9:** Comparison of estimated values and experimental values based on area.

Group Number	Strength			Modulus		
	Estimated Value/MPa	Experimental Value/MPa	Error/%	Estimated Value/MPa	Experimental Value/MPa	Error/%
W1	115.90	113.78	1.87%	10,696.63	10,401.64	2.84%
W2	97.26	94.03	3.44%	9886.38	9584.51	3.15%
W3	97.26	89.63	8.52%	9886.38	9093.96	8.71%
W4	73.75	73.22	0.72%	9215.00	9493.27	−2.93%
W5	103.15	109.68	−5.95%	10,092.43	10,998.01	−8.23%
W6	103.15	106.53	−3.17%	10,092.43	10,766.68	−6.26%
W7	90.78	94.27	−3.70%	9684.55	9193.00	5.35%
Average			0.24%			0.37%

**Table 10:** Specific strength and modulus results.

Group Number	Statistical Parameters	Specific Strength/×10^−3^ MPa·kg^−1^·m^3^	Specific Modulus/×10^−3^ MPa·kg^−1^·m^3^	Density/×10^3^ kg·m^−3^
L1	Mean value	178.75	16,239.25	0.64
	Standard deviation	22.43	2633.95	0.05
	CV	12.55%	16.22%	8.33%
L2	Mean value	159.87	16,403.40	0.59
	Standard deviation	27.15	2453.60	0.03
	CV	16.98%	14.96%	5.03%
L3	Mean value	146.91	14,845.54	0.61
	Standard deviation	32.64	1259.04	0.04
	CV	22.22%	8.48%	5.80%
L4	Mean value	115.93	14,949.45	0.63
	Standard deviation	28.29	1196.43	0.03
	CV	24.40%	8.00%	4.63%
L5	Mean value	174.72	17,481.72	0.63
	Standard deviation	29.44	1820.60	0.04
	CV	16.85%	10.41%	6.73%
L6	Mean value	170.39	17,300.44	0.63
	Standard deviation	13.97	1198.35	0.04
	CV	8.20%	6.93%	6.32%
L7	Mean value	155.11	15,129.59	0.61
	Standard deviation	19.44	1640.01	0.05
	CV	12.53%	10.84%	8.24%

**Table 11:** Parameters of size effect models based on method 2.

Properties	*D* Index	Weakest-Link Model			Fractural Model			Fractal Theory Model		
		A_1_	B_1_	SSE	B_2_	D_0_	SSE	A_3_	B_3_	SSE
Specific strength	Thickness	307.00	0.30	324.90	250.00	6.09	215.40	8840.00	1.39 × 10^5^	481.30
	Area	388.40	0.18	632.70	204.40	220.10	326.80	1.64 × 10^4^	1.02 × 10^6^	1147.00
Specific modulus	Thickness	1.99 × 10^4^	0.096	4.74 × 10^6^	1.61 × 10^4^	−2.17 × 10^7^	7.28 × 10^6^	2.08 × 10^8^	4.27 × 10^8^	5.13 × 10^6^
	Area	2.15 × 10^4^	0.059	5.08 × 10^6^	1.61 × 10^4^	−4.41 × 10^8^	7.28 × 10^6^	2.36 × 10^8^	2.66 × 10^9^	6.10 × 10^6^

**Table 12:** Parameters of size effect models based on method 3.

Properties	*D* index	Weakest-Link Model			Fractural Model			Fractal Theory Model		
		A_1_	B_1_	SSE	B_2_	D_0_	SSE	A_3_	B_3_	SSE
Strength	Thickness	301.80	0.30	120.00	245.50	6.40	77.52	9079.00	1.35 × 10^5^	181.20
	Area	380.50	0.18	235.00	202.60	226.80	116.90	1.65×10^4^	9.90 × 10^5^	432.90
Modulus	Thickness	1.95 × 10^4^	0.089	1.61 × 10^6^	1.60 × 10^4^	−1.38 × 10^7^	2.47×10^6^	2.11 × 10^8^	3.95 × 10^8^	1.72 × 10^6^
	Area	2.10 × 10^4^	0.055	1.71 × 10^6^	1.60 × 10^4^	−2.96 × 10^8^	2.47×10^6^	2.36 × 10^8^	2.51 × 10^9^	2.04 × 10^6^

**Table 13:** Estimated values and experimental values based on method 2.

*D* Index	Group Number	Estimated Specific Strength/×10^−3^ MPa·kg^−1^·m^3^	Strength			Estimated Specific Modulus/×10^−3^ MPa·kg^−1^·m^3^	Modulus		
			Estimated Value/MPa	Experimental Value/MPa	Error/%		Estimated Value/MPa	Experimental Value/MPa	Error/%
Thickness	W1	185.29	118.65	113.78	4.28%	17,004.96	11,223.27	10,401.64	7.90%
	W2	153.84	90.54	94.03	−3.72%	15,908.91	9362.71	9584.51	−2.31%
	W3	153.84	93.87	89.63	4.73%	15908.91	9707.43	9093.96	6.75%
	W4	120.82	75.98	73.22	3.76%	14,883.51	9525.45	9493.27	0.34%
	W5	167.39	105.63	109.68	−3.69%	16,354.97	10,321.10	10,998.01	−6.15%
	W6	167.39	104.67	106.53	−1.75%	16,354.97	10,467.18	10,766.68	−2.78%
	W7	153.84	94.06	94.27	−0.22%	15,908.91	9863.53	9193.00	7.29%
	Average				0.48%				1.58%
Area	W1	184.51	118.15	113.78	3.84%	17,056.11	11,257.03	10,401.64	8.22%
	W2	157.63	92.77	94.03	−1.35%	15,986.51	9408.38	9584.51	−1.84%
	W3	157.63	96.18	89.63	7.31%	15,986.51	9754.78	9093.96	7.27%
	W4	121.78	76.58	73.22	4.59%	15,088.39	9656.57	9493.27	1.72%
	W5	166.28	104.93	109.68	−4.33%	16,259.94	10,261.13	10,998.01	−6.70%
	W6	166.28	103.98	106.53	−2.40%	16,259.94	10,406.36	10,766.68	−3.35%
	W7	147.95	90.46	94.27	−4.04%	15,717.68	9744.96	9193.00	6.00%
	Average				0.52%				1.62%

**Table 14:** Comparison of estimated values and experimental values based on method 3.

*D* Index	Group Number	Strength			Modulus		
		Estimated Value/MPa	Experimental Value/MPa	Error/%	Estimated Value/MPa	Experimental Value/MPa	Error/%
Thickness	W1	117.77	113.78	3.51%	11,154.27	10,401.64	7.24%
	W2	90.24	94.03	−4.04%	9353.91	9584.51	−2.41%
	W3	93.56	89.63	4.38%	9698.30	9093.96	6.65%
	W4	75.99	73.22	3.78%	9566.36	9493.27	0.77%
	W5	105.10	109.68	−4.17%	10,289.05	10,998.01	−6.45%
	W6	104.15	106.53	−2.24%	10,434.67	10,766.68	−3.08%
	W7	93.75	94.27	−0.55%	9854.25	9193.00	7.19%
	Average			0.10%			1.42%
Area	W1	117.43	113.78	3.21%	11,190.50	10,401.64	7.58%
	W2	92.51	94.03	−1.62%	9395.41	9584.51	−1.97%
	W3	95.91	89.63	7.01%	9741.33	9093.96	7.12%
	W4	76.64	73.22	4.67%	9682.49	9493.27	1.99%
	W5	104.53	109.68	−4.69%	10,234.79	10,998.01	−6.94%
	W6	103.58	106.53	−2.77%	10,379.64	10,766.68	−3.59%
	W7	90.31	94.27	−4.21%	9743.13	9193.00	5.98%
	Average			0.23%			1.45%

## Data Availability

The original contributions presented in this study are included in the article. Further inquiries can be directed to the corresponding authors.
